# Double Ovariotomy at the Age of Sixty-Nine Years

**Published:** 1888-08

**Authors:** E. J. Beall

**Affiliations:** Fort Worth, Texas


### DOUBLE OVARIOTOMY AT THE AGE OF SIXTY-NINE YEARS.

### Tumors Weighing, Respectively, Eleven and Thirty-Six Pounds.

BY E. J. BEALL, M. D., FORT WORTH, TEXAS.

For Daniel's Texas Medical Journal.

JULY 16, 1888, I did a double ovariotomy upon a lady, residing in this city, who was sixty-nine years of age the sixth of August, The combined weight of the tumors was forty-seven pounds.

The adhesions were considerable, necessitating fifteen ligatures within the abdominal cavity.

My personal experience in abdominal surgery, and observation in that line of work, furnish no case that progressed more readily to complete recovery, It proceeded without an untoward symptom.

I think the case tends to confirm a remark of my distinguished friends, Drs. Wooten and Leake, "that old age should not debar one from the benefit of our art."

Will some of your readers refer to a *double* ovariotomy in a subject older than the one I here record?

I did a lithotomy quite recently upon a gentleman nearly seventy years of age, removing two calculi. Recovery without a bad symptom.

